# The role of cortical structural variance in deep learning-based prediction of fetal brain age

**DOI:** 10.3389/fnins.2024.1411334

**Published:** 2024-05-23

**Authors:** Hyeokjin Kwon, Sungmin You, Hyuk Jin Yun, Seungyoon Jeong, Anette Paulina De León Barba, Marisol Elizabeth Lemus Aguilar, Pablo Jaquez Vergara, Sofia Urosa Davila, P. Ellen Grant, Jong-Min Lee, Kiho Im

**Affiliations:** ^1^Department of Electronic Engineering, Hanyang University, Seoul, Republic of Korea; ^2^Fetal Neonatal Neuroimaging and Developmental Science Center, Boston Children’s Hospital, Boston, MA, United States; ^3^Division of Newborn Medicine, Boston Children’s Hospital, Boston, MA, United States; ^4^Department of Pediatrics, Harvard Medical School, Boston, MA, United States; ^5^Department of Radiology, Harvard Medical School, Boston, MA, United States; ^6^Department of Biomedical Engineering, Hanyang University, Seoul, Republic of Korea; ^7^Department of Artificial Intelligence, Hanyang University, Seoul, Republic of Korea

**Keywords:** magnetic resonance imaging, deep learning, fetal brain age, cortical surface, sulcal pattern

## Abstract

**Background:**

Deep-learning-based brain age estimation using magnetic resonance imaging data has been proposed to identify abnormalities in brain development and the risk of adverse developmental outcomes in the fetal brain. Although saliency and attention activation maps have been used to understand the contribution of different brain regions in determining brain age, there has been no attempt to explain the influence of shape-related cortical structural features on the variance of predicted fetal brain age.

**Methods:**

We examined the association between the predicted brain age difference (PAD: predicted brain age–chronological age) from our convolution neural networks-based model and global and regional cortical structural measures, such as cortical volume, surface area, curvature, gyrification index, and folding depth, using regression analysis.

**Results:**

Our results showed that global brain volume and surface area were positively correlated with PAD. Additionally, higher cortical surface curvature and folding depth led to a significant increase in PAD in specific regions, including the perisylvian areas, where dramatic agerelated changes in folding structures were observed in the late second trimester. Furthermore, PAD decreased with disorganized sulcal area patterns, suggesting that the interrelated arrangement and areal patterning of the sulcal folds also significantly affected the prediction of fetal brain age.

**Conclusion:**

These results allow us to better understand the variance in deep learning-based fetal brain age and provide insight into the mechanism of the fetal brain age prediction model.

## 1 Introduction

Existing neuroimaging studies have shown that brain age estimated using magnetic resonance imaging (MRI) can serve as an imaging biomarker for assessing brain health at the individual level (Cole et al., 2017). The estimated brain age is considered the biological or neuroanatomical age, which may differ from the chronological age (He et al., 2021b). Deviations in the predicted age from the chronological age can reveal potential underlying pathological processes in the brain (Hong et al., 2021; He et al., 2021a). Numerous previous studies have shown that the predicted age difference (PAD; predicted brain age–chronological age) is associated with the risk of cognitive decline and neurodegeneration in various disorders of the neonatal brain (Liu et al., 2020, 2021) and the adult brain (Schnack et al., 2016; Ning et al., 2020). The fetal brain undergoes dramatic anatomical changes during development under genetic influences (Andescavage et al., 2017). As a result of brain development, quantitative structural brain measures including fetal brain volume, surface area, folding pattern, and gyrification were strongly correlated with gestational age (GA; Andescavage et al., 2017). The estimation of fetal brain age and its deviation from GA based on MRI morphological patterns is a potentially useful tool for identifying early brain abnormalities and improving prenatal care (Liao et al., 2020).

Recent advances in deep learning algorithms have enabled accurate mapping between fetal MRI and GA. Shi et al. (2020) used a convolutional neural network (CNN)-based model to predict the fetal brain age using MRI. Gangopadhyay et al. (2022) utilized a multitasking U-Net with a single encoder to predict the fetal brain age and pathological conditions simultaneously. Previous studies have improved fetal brain age prediction by applying attention-guided CNN (Shen et al., 2022), 2D multislice augmented CNN (Hong et al., 2021), and label distribution learning (Liao et al., 2020) to fetal MRI. However, unlike previous studies that have directly associated brain features with age, deep CNN-based brain age models remain challenging to interpret because they utilize highly non-linear functions for prediction. It remains difficult to explain how structural brain changes affect brain-age predictions. Recent approaches for interpreting deep learning-based brain age can be divided into two categories. First, a gradient-based mechanism was employed to identify regions that significantly influenced brain age prediction by mapping salient features to the raw input image. In fetal brain age studies, the most contributing regions have been identified using Grad-CAM (Liao et al., 2020), back-propagation (Hong et al., 2021), and guided back-propagation (Shi et al., 2020). Methods in the second category utilize attention mechanisms that emphasize global and local features to predict brain age and generate interpretations simultaneously. Shen et al. (2022) used an attention-guided mask to provide heat maps that highlighted the most contributing regions. Shi et al. (2020) generated attention activation maps using multiscale features from networks in addition to saliency maps. Although the saliency map and attention heat map help us understand localized explanations for brain age prediction without prior knowledge, they only show the local region where and what the network learns, meaning that they provide an indirect interpretation (He et al., 2021a). Our understanding of which specific brain structural features and regions significantly contribute to the variance in predicted brain age and how they contribute remains limited. To the best of our knowledge, correlations between regional brain volumes and predicted brain age have been investigated in healthy brains with ages ranging from 0 to 97 years, but the results were not statistically significant (He et al., 2021a). In fetal brains, our CNN-based prediction model demonstrated that whole brain size significantly affects brain age, and saliency maps showed that cortical regions play an important role in predicting fetal brain age (Hong et al., 2021). However, despite the potential significance of cortical structures in brain age prediction, investigation of the relationship between predicted brain age and human-understandable morphological characteristics of the brain surface model is still limited. The morphological changes in fetal brains during gestation are complex and various. As the fetal brain grows, cortical areas get larger, increasing the depth and complexity of developing cortical foldings. Even though the saliency map helps the interpretation of the predicted brain age by indicating related brain regions, no study has investigated which aspect of those complex changes in brain development affects brain age estimation.

**Figure 1:**
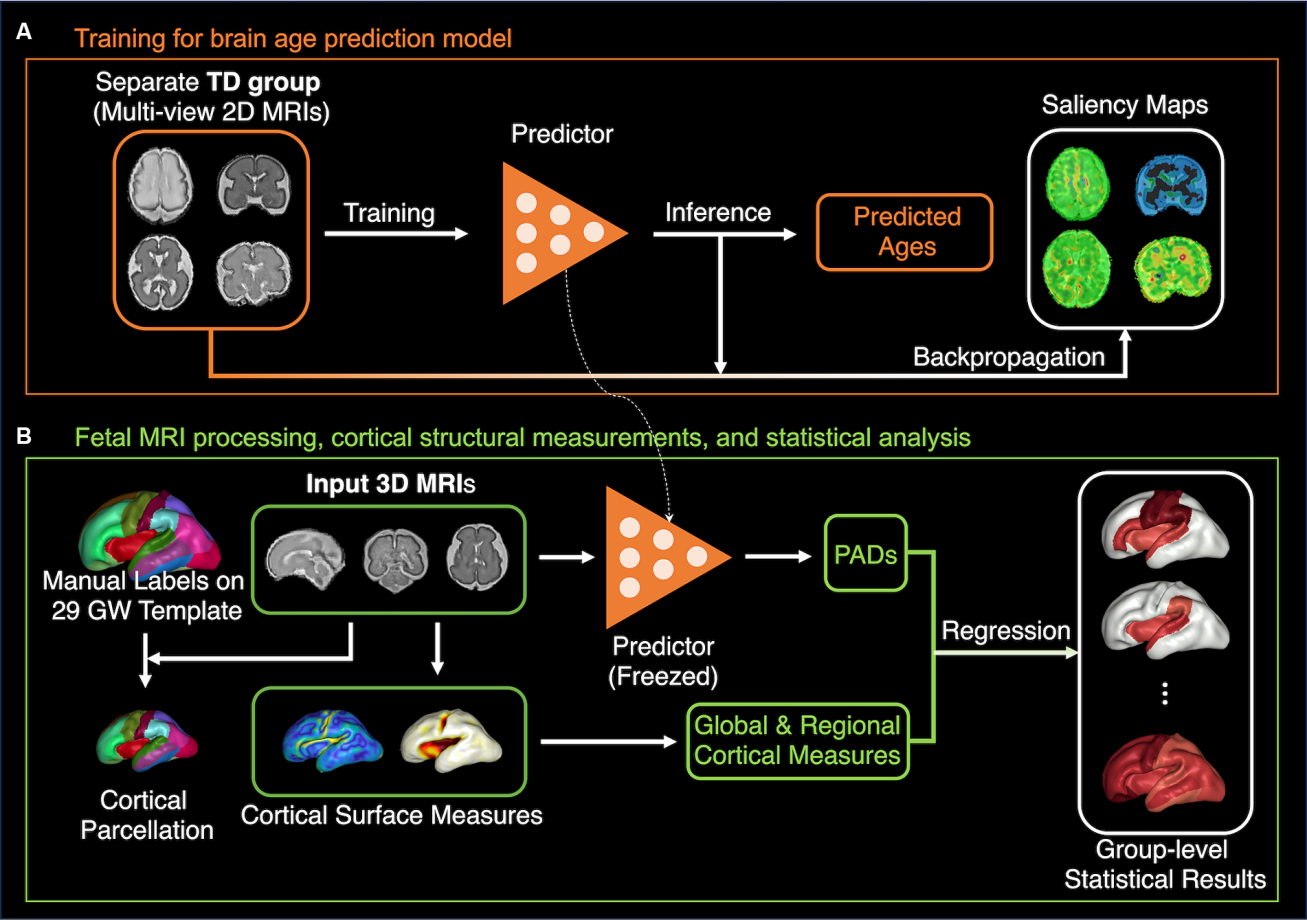
The pipeline of method and analysis. **(A)** The brain age prediction model based on ResNet101-V2 is trained with separate TD fetal data. The saliency maps for each individual 2D MRI sample are calculated. **(B)** The relationships between the PAD from the model in panel **(A)** and global and regional cortical measurements were examined using the regression analysis.

Given the scenario, this study aimed to investigate the association between deep-learning-based brain age predictions and the global/regional cortical measurements to understand which features and how they contribute to brain age predictions. We utilized a single-channel CNN with multiplanar slices as proposed in a previous study on PAD estimation (Hong et al., 2021). We then calculated various global and regional cortical structural features, such as cortical volume, surface area, curvature, gyrification index (GI), and sulcal depth, and analyzed their correlations with the estimated PAD using a regression model. Furthermore, we examined the effect of global sulcal folding patterns on PAD. The saliency map method was used to evaluate and compare correlation results. The study design is illustrated in Figure 1.

## 2 Materials and methods

### 2.1 Subjects

A total of 115 typically developing (TD) fetuses were included in this study (gestational weeks [GW]: 29.4 ± 4.4 [mean ± standard deviation (SD)], range: 19.9–38.7 GW; sex: 57/42/16 [male/female/unknown]). We included fetuses with a maternal age of 19.0–43.3 years (32.5 ± 4.5 [mean ± SD]). Subjects demographics including *n*, sex ratio, and maternal age along with GA segments are summarized in Table 1. For the quantitative sulcal pattern analysis, fetuses younger than 23 GW or older than 32 GW were excluded because we employed template brains ranging from 23 to 32 GWs for sulcal pattern matching and similarity measurement. Subsequently, 76 fetuses from 23 to 32 GW were included in the quantitative sulcal pattern analysis. To test the interaction between measure and sex, 16 of the 115 fetuses were excluded because their sex was unknown.

**Table 1:** Participant characteristics across gestational age segments.

	<24 GW	24–28 GW	28–32 GW	32–36 GW	≥36 GW	Total
*n* (male/female/unknown)	7/4/0	17/18/4	18/8/6	6/8/5	9/4/1	57/42/16
Maternal age	28.9 ± 5.2 (20.0–35.0)	31.7 ± 4.9 (19.0–40.0)	34.2 ± 3.9 (28.0–43.3)	33.0 ± 3.6 (26.6–39.0)	32.7 ± 3.2 (27.7–38.0)	32.5 ± 4.5 (19.0–43.3)

GW, gestational weeks.Mean ± standard deviation (min-max range) for Maternal age (years) are represented.

### 2.2 Data and image acquisition

This study was approved by the Institutional Review Board of Boston Children’s Hospital. TD subjects were identified from (1) prospective recruitment subjects for case–control studies who signed written informed consent, and (2) retrospective patient data that were screened for fetal brain abnormalities but were clinically interpreted as normal by two board-certified radiologists experienced in fetal MRI. We excluded women with multiple gestational pregnancies, dysmorphic features on ultrasound (US) examination, brain malformations/lesions or other identified organ anomalies on US examination, known chromosomal abnormalities, known congenital infections, or any clinically significant abnormalities on visual inspection. Fetal brain MRI stacks were acquired on a Siemens 3 T Skyra scanner using a T2-weighted HASTE (Half-Fourier Acquisition Single-Shot Turbo Spin-Echo) sequence with 1 mm in-plane resolution, FOV = 256 mm, time repetition = 1.5 s, time echo = 120 ms, and slice thickness = 2–4 mm. After localization to the fetal brain, a total of 3–20 HASTE stacks were acquired multiple times in three different orthogonal orientations for reliable image processing and analysis (the scan time for acquisition of MRI stacks was 10–20 min).

### 2.3 Fetal MRI pre-processing and surface reconstruction

We used a previously developed pipeline for fetal MRI pre-processing and inner cortical plate surface extraction (Yun et al., 2021, 2024). The brain region was masked using our in-house tool based on a 2D U-Net model for each MRI stack (Hong et al., 2021). N4 bias field correction was used to correct the intensity non-uniformity of the masked brain (Tustison et al., 2010). Multiple MRI stacks were combined using a slice-to-volume registration technique and reconstructed a motion-corrected volume with 0.75 mm isotropic super-resolution (Kuklisova-Murgasova et al., 2012). We then adopted a deep learning-based approach to segment the cortical plate and its inner part (Hong et al., 2020). We automatically extracted the hemispheric triangular surface meshes of the inner cortical plate using a marching cube algorithm with topology preservation (Lepage et al., 2017). The automatic cortical segmentation results were visually inspected, and any mislabeled regions were manually edited by trained raters to validate the quality of cortical surface extraction and measurement.

### 2.4 Brain age prediction and PAD estimation

The brain age prediction model (Hong et al., 2021) was built based on ResNet101V2 (He et al., 2016) with slight modifications to reduce model complexity and prevent overfitting by replacing the last pooling layer with global average pooling and adding a dropout layer (dropout rate = 0.4). A 2D slice of brain MRI with a size of 138 × 176 was used as the input for the network, creating the last feature maps with a reduced size of 5 × 6 via stacked residual blocks, which were connected to a dense layer to regress the predicted brain age. The detailed implementation and hyperparameter settings were the same as those of the original ResNet101V2, The batch size was set to 128, and the Adam optimizer (Kingma and Ba, 2014)with a learning rate of 0.05 was used. We used the Huber loss as a loss function, which is less sensitive to outliers (Huber, 1992). For the training of the brain age prediction model, we used 7,156 slices from 1,789 MRI volumes from separate 136 TD fetuses (GW: 30.0 ± 5.5 [mean ± SD], range: 15.9–38.7 GW; sex: 58/37/41 [male/female/unknown]) with the subject-wise random split (Figure 1A). Among the training sets, we used 5,696 slices from 1,424 MRI volumes recorded from 111 fetuses for optimization, with four central slices from each MRI volume. The remaining 1,460 slices from 365 MRI volumes recorded from other 25 fetuses, were used for validation during training.

In this study, no strategy for data splitting, such as cross-validation, was used because the performance of our brain age prediction was evaluated in a previous study (Hong et al., 2021) which focused on the analysis of the relationship between the estimated PAD and cortical structural features from fetal brain MRI. The brain age prediction model used in this study has proven the effectiveness of precise brain age prediction via multi-view aggregation with central tendency estimation showing accurate prediction compared to the 2D multi-channel or 3D approach (Hong et al., 2021). Using central four slides as input helps the model clearly focus on the distinctive regions for brain age prediction, such as the ventricles. Besides, considering the thickness of fetal MRI, the four central slides on three different planes (sagittal, axial, and coronal) could cover most anterior–posterior, superior–inferior, and left–right structures of the fetal brain within the model’s receptive field.

For the brain age prediction and PAD estimation in the target dataset, we applied our in-house fetal brain extraction tool and N4 bias field correction (Tustison et al., 2010; Hong et al., 2020) on the raw fetal MRI before performing brain age estimation on them. We selected central four slices for each volume as the inputs for the brain age prediction model and performed test-time augmentation (Matsunaga et al., 2017) with 20 repetitions to minimize the prediction error by ensembling multiple predictions with augmentations for each slice. We then computed the mode for continuous variables (Pearson, 1894) to obtain the central tendency of the brain age prediction from multiple predicted age values with multiple volumes and slices as the estimated brain age for each case. Finally, we measured the difference between the predicted brain age and the corresponding GA to estimate the PAD for each case.

### 2.5 Cerebral volume and global cortical surface measures

**Figure 2:**
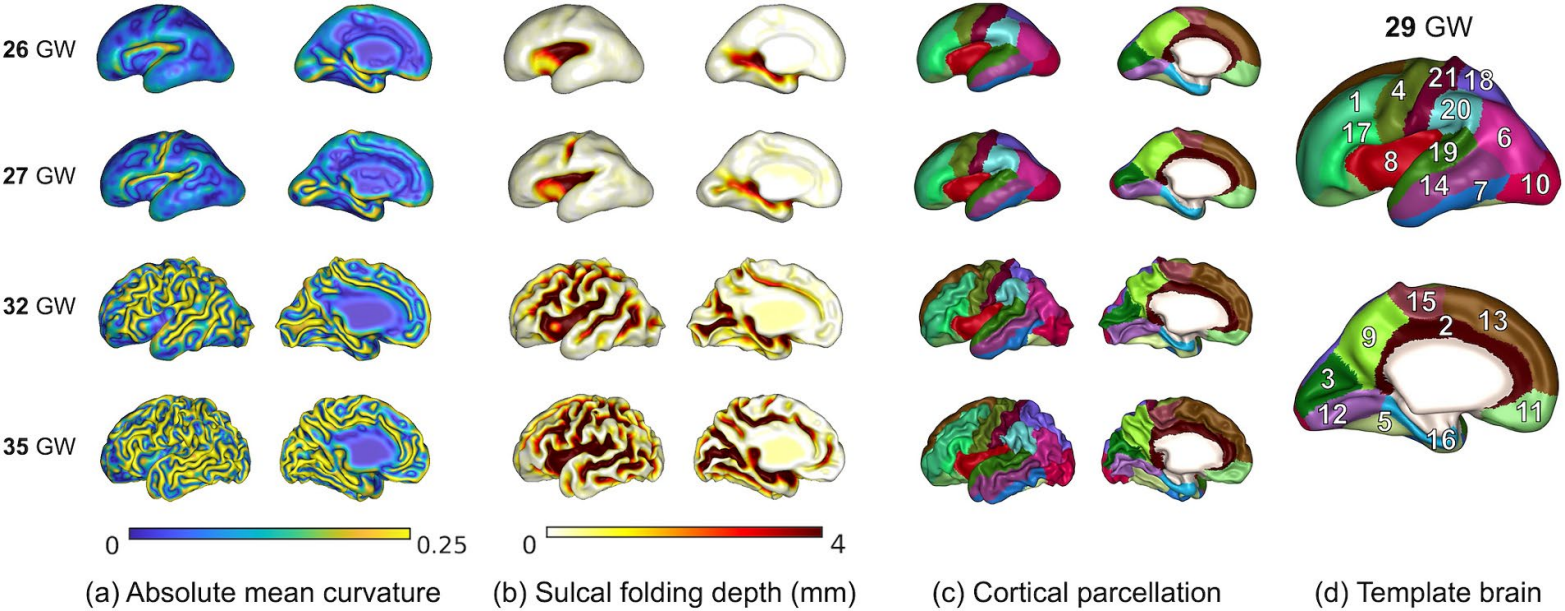
Mapping of absolute mean curvature **(A)**, sulcal depth **(B)**, and cortical parcellation **(C)** on the cortical surface for 4 individual fetuses of 26 to 35 GW and 29 GW template brain **(D)**. Anatomical labels: 1. middle frontal, 2. cingulate cortex, 3. cuneus, 4. precentral, 5. fusiform, 6. inferior parietal, 7. inferior temporal, 8. insula, 9. precuneus, 10. lateral occipital, 11. orbital frontal, 12. lingual, 13. superior frontal, 14. middle temporal, 15. paracentral, 16. parahippocampal, 17. inferior frontal, 18. superior parietal, 19. superior temporal, 20. supramarginal, 21. postcentral.

We measured the cerebral volume, cortical surface area, average absolute mean curvature, GI, and average sulcal depth of the whole brain (Figure 1B). The surface area of each vertex was computed using the area of the Voronoi region (Meyer et al., 2003). The angular deviation from the patch around each vertex was calculated as the mean curvature. Positive and negative signs of the mean curvature indicate outwardly and inwardly folded regions, respectively (Meyer et al., 2003). We used the absolute mean curvature to measure the complexity of the cortical folding shape (Figure 2A). To define the GI, we first performed a 3D morphological closing operation with a spherical kernel of 15 mm diameter as a structural element on the inner volume of the cortical plate (Schaer et al., 2008). Using the marching cube algorithm, we created the outer hull surface wrapping the cortical plate surface from the binary closed volume (Lepage et al., 2017). The ratio between the 3D convex hull and the entire area of the cortical plate surface was then calculated (Zilles et al., 1988). Sulcal depth was calculated using our adaptive distance transform, which searches for the shortest paths from the convex hull to the surface vertices (Yun et al., 2013; Figure 2B). The global cortical surface measures were correlated with GA and the changes in those measurements along with GA were visually inspected to ensure the reliability of results. Specifically, cortical surfaces were identified as outliers if they exhibited the deviation from typical developmental patterns in global cortical measures along with GA.

### 2.6 Cortical parcellation and regional measures

We manually parcellated and defined the cortical gyral regions on the 29 GW template surface according to the FreeSurfer Desikan parcellation protocol, which has been extensively used as a standard in neuroimaging studies (Desikan et al., 2006). The template surface was extracted from the previously generated T2 MRI volume templates (Serag et al., 2012). The original Desikan parcellation map includes 34 cortical regions in each hemisphere. As the secondary and tertiary sulci are not fully developed in the fetal cortex, it is not feasible to parcellate the subdivisions of the gyrus. The original map was simplified and 21 cortical areas were delineated in each hemisphere. Individual cortical plate surfaces were aligned to the template surface using a 2D sphere-to-sphere warping method (Robbins et al., 2004; Boucher et al., 2009) and then resampled to obtain the vertex correspondence with the template. The parcellated regions on the template were directly applied to the registered individual surfaces (Figures 2C,D), and the cortical surface area, average absolute mean curvature, and average sulcal depth for each cortical region were calculated (Figure 1B).

### 2.7 Quantitative sulcal pattern analysis

To investigate the relationship between the estimated PAD and sulcal folding patterns, we performed a quantitative sulcal pattern analysis proposed by Im et al. (2011, 2017). We first identified the sulcal basins based on a smoothed curvature map of the extracted cortical surface using a watershed algorithm (Tarui et al., 2018). Spectral matching was performed using geometric sulcal features (3D position, depth, and area) to define the correspondence between the sulcal basins of an individual brain and those of the fetal template brain (Im et al., 2017). For each fetus, the sulcal pattern similarity index (SI) of the templates, reflecting the deviation from the typical sulcal pattern, was defined by averaging the similarities of all matched corresponding sulcal basins and inter-sulcal relationships (Tarui et al., 2023). We assessed the pattern similarities of 3D sulcal position, sulcal area, sulcal depth, and a combination of all three features by changing the weight of each feature (Ortinau et al., 2019). Additionally, we separately calculated the similarities of the corresponding sulcal regions and the inter-sulcal geometric relationships between the individual and template brains.

### 2.8 Statistical analysis

We analyzed how the regional cortical structural changes affect the estimated PAD using a linear regression model, adjusting for GW ($\mathrm{PAD}=\beta_{0}+\beta_{1}GW+\beta_{2}X$, where X denotes global and regional cortical measurements or sulcal pattern SI; Figure 1B). We assessed the association between the estimated PAD and global and regional cortical measurements calculated from the extracted surface models for each hemisphere. Sulcal pattern SIs to the template brains were also used to examine their effect on PAD using the same regression model. Additionally, we examined the measure-by-sex interaction effect to test statistically whether sex influenced the relationship between PAD and cortical structural features. A false discovery rate (FDR) control was used at a q value of 0.05 to correct for multiple comparisons (Benjamini and Hochberg, 1995).

### 2.9 Saliency map

We generated a saliency map to assign contribution scores to each input element (e.g., pixels in an input 2D MRI slice) for deep CNN-based brain-age prediction (Figure 1A). We used the back-propagation method to track the brain regions that exhibited a high contribution to brain age prediction. The resulting saliency maps were also Gaussian-smoothed and min-max normalized for better visibility, as proposed in a previous study (Hong et al., 2021). We then identified areas with high saliency scores and visually compared them with areas that showed a significant correlation with brain age in our regression analysis.

## 3 Results

Before analyzing the relationship between PAD and cortical surface measurement, we first confirmed our model’s brain age prediction performance after test-time augmentation and central tendency estimation via mode. Our brain age prediction model showed a mean absolute error (MAE) of 0.94 GW and $R^{2}$ of 0.908 for the target dataset which was used for the analysis in this study.

As shown in Table 2, a significant positive correlation between whole cerebral volume and estimated PAD was observed in the regression analysis (*p* < 0.0001). The cortical surface area (*p* = 0.0005) and absolute mean curvature (*p* = 0.047) of the whole brain were significantly increased in fetuses with higher PAD. No significant relationships were found between the global cortical GI (*p* = 0.341) and sulcal depth (*p* = 0.178).

**Table 2:** Statistical results of the regression analysis investigating the association between the estimated PAD and global measurements.

Measures	*β* ± *SE* (*p-*value)
Whole cerebral volume	1.970 ± 0.334 (<0.0001^**^)
Cortical surface area	1.133 ± 0.316 (0.0005^**^)
Cortical mean curvature	0.717 ± 0.356 (0.047^*^)
Cortical GI	0.216 ± 0.226 (0.341)
Cortical depth	0.336 ± 0.248 (0.178)

β, standardized regression coefficient; SE, standard error; GI, gyrification index.^**^p < 0.0005.^*^p < 0.05.

**Figure 3:**
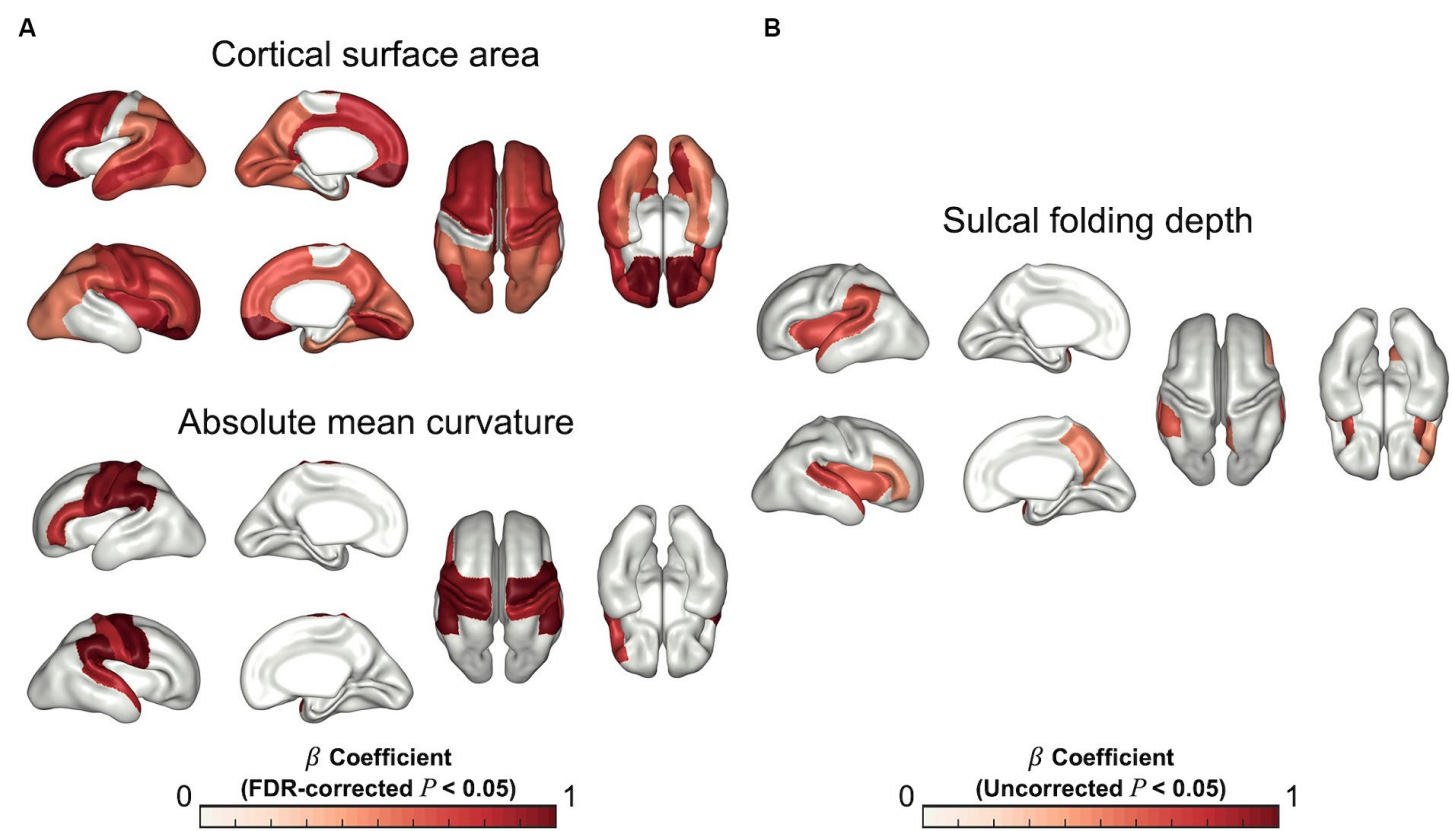
Statistical results for regression analysis of regional cortical measurements. Statistical maps for *β* coefficients show positive correlations between the estimated PAD and cortical surface area and absolute mean curvature **(A)** (FDR-corrected *p* < 0.05), and sulcal folding depth **(B)** (Uncorrected *p* < 0.05).

In the regression analysis of the regional cortical measurements, fetuses with higher PAD showed significantly higher cortical surface areas in most cortical regions, except for the paracentral and parahippocampal cortices after FDR correction (Figure 3A). The regional cortical curvatures were positively correlated with PAD in the precentral (*p* < 0.001), postcentral (*p* < 0.001), cuneus (*p* = 0.011), supramarginal (*p* < 0.001), superior temporal (*p* = 0.013), and inferior frontal (*p* = 0.009) cortices (Figure 3A). There were no significant correlations in the analysis of sulcal depth after FDR correction. However, the insula (uncorrected *p* = 0.007), precuneus (uncorrected *p* = 0.029), superior temporal (uncorrected *p* = 0.008), and supramarginal cortical regions (uncorrected *p* = 0.028) were significantly positively correlated with PAD (Figure 3B). The details of the statistical results are provided in Supplementary Table S1.

Statistically significant associations were observed between sulcal pattern similarity and the estimated PAD for the sulcal basin area in the entire pattern (*p* = 0.005) and inter-sulcal relationship (*p* = 0.020) analyses (Table 3). Subjects with higher PAD showed a higher sulcal area pattern similarity to the normal templates. In the case of sulcal position and depth patterns, there was no significant relationship between sulcal pattern similarity and estimated PAD. The association between the combinations of all three features was not statistically significant.

**Table 3:** Statistical results of the regression analysis investigating the association between the estimated PAD and sulcal pattern similarity.

Sulcal pattern similarity	Position	Depth	Area	Combined
Whole pattern	−0.039 ± 0.156 (0.804)	0.121 ± 0.137 (0.382)	0.413 ± 0.143 (0.005*)	0.133 ± 0.131 (0.311)
Corresponding sulcal regions	−0.008 ± 0.127 (0.948)	0.124 ± 0.152 (0.419)	0.219 ± 0.123 (0.078)	0.157 ± 0.116 (0.180)
Inter-sulcal relationship	−0.010 ± 0.150 (0.947)	0.129 ± 0.133 (0.334)	0.300 ± 0.126 (0.020^*^)	0.092 ± 0.144 (0.523)

Data are represented as β ± SE (*p*-value).^*^p < 0.05.

**Figure 4:**
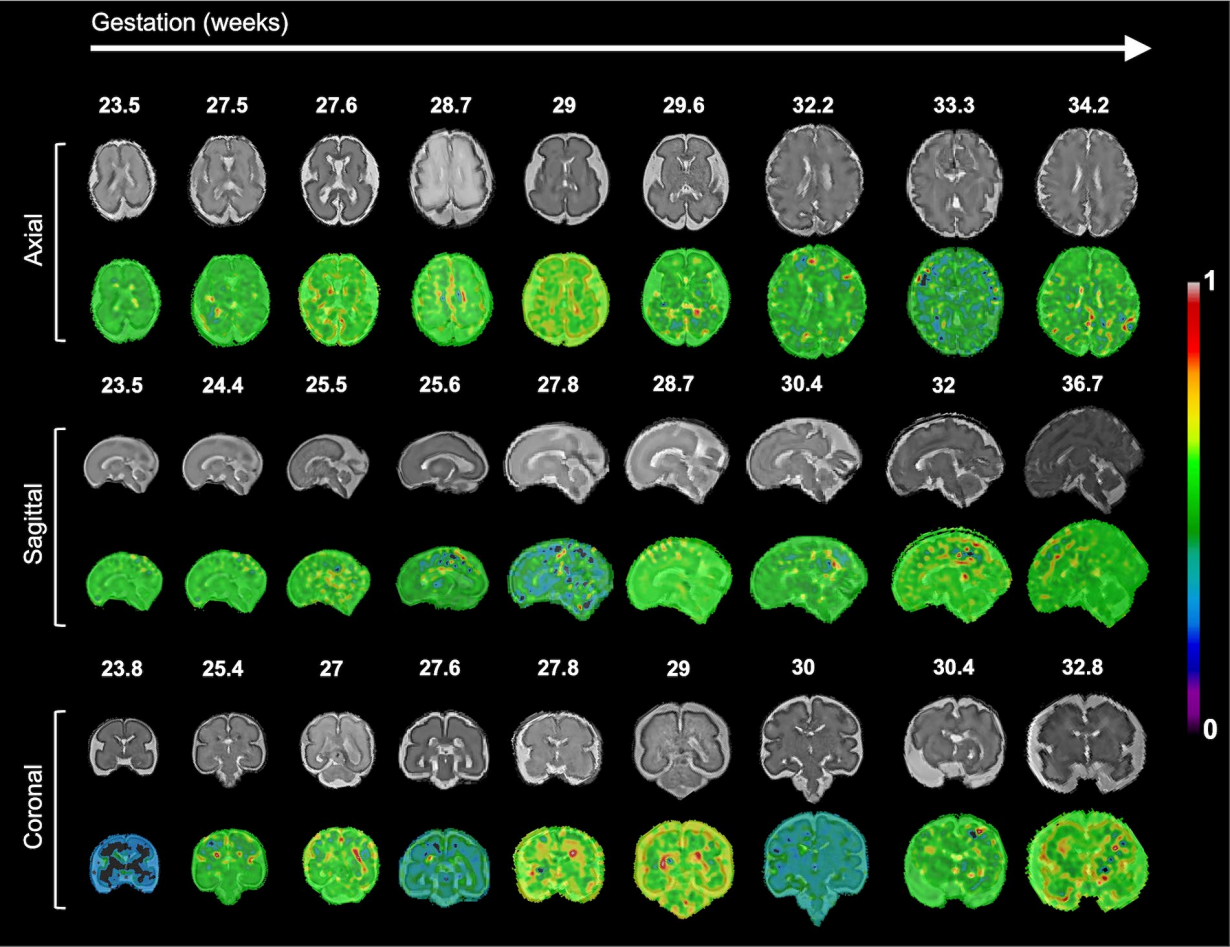
Examples of the saliency map for deep CNN-based brain age prediction model on input 2D MRI slices. The high saliency values indicate that those regions most contribute to the brain age prediction.

The resulting saliency maps for the randomly selected 2D MRI samples are shown in Figure 4. We observed that specific regions, including the precentral, postcentral, (pre)cuneus, supramarginal, superior temporal, and inferior frontal cortices showed high saliency values, which were significantly associated with PAD in our regression analysis. However, beyond the overlapping areas, regions with high saliency scores showed large spatial variability across individuals, precluding meaningful group-level interpretations.

No significant interaction effects of sex were detected in any of the global or regional brain measures.

## 4 Discussion

We examined the association between the estimated PAD on fetal brain MRI and global and regional cortical structural features, such as whole cerebral volume, cortical surface area, curvature, GI, folding depth, and sulcal patterns. Although existing deep-learning-based brain age prediction studies have employed either input-level salience maps or attention mechanisms and have shown a localized visual explanation without any prior domain-specific knowledge, they are limited by the inherent ambiguity of indirect interpretations. In contrast, Hong et al. (2021) showed that the cerebral cortex contributed significantly to estimating brain age by applying saliency visualization to their prediction model. Furthermore, they replicated the brain age prediction after adjusting for the brain size of individuals and suggested that whole brain size could considerably affect the predicted brain age. Motivated by these findings, we examined the relationship between these cortical structural changes and estimated PAD. Given that regulated areal expansion and folding of the human brain cortex occurs during fetal development, complicated patterns of cortical development, such as curvature, GI, depth, and sulcal patterns of the cortex, have been characterized (Im et al., 2017). Our results showed significant relationships between these cortical structural variances and the estimated PAD in global and local cortical regions. Significant regions, including the precentral, postcentral, (pre)cuneus, supramarginal, superior temporal, and inferior frontal cortices were also identified as contributing regions in the saliency map analysis. However, unlike our results, the saliency maps were only acquired from a limited region of a few central 2D MRI slices, lacking comprehensive information. The saliency maps highlighted the contributing regions without offering any rationale for the prediction related to brain structural changes, whereas the proposed method demonstrated associations between various global and local cortical structural variances and the predicted PAD. Moreover, the most contributive regions in the saliency map exhibited a large inter-subject spatial variability as shown in Figure 4, which can lead to vague group-level explanations. In contrast, our results provide a better interpretation by utilizing complicated structural features derived from whole-brain cortical surfaces within the training dataset of the prediction model.

Several studies have explored the non-deep-learning-based relationship between fetal cortical folding features and GA. Clouchoux et al. (2012) characterized fetal cortical folding features, such as the sulcal area and GI, and suggested a non-linear relationship between the measures and GA. Wu et al. (2015) applied a regression model to predict the GA using eight different sulcal folding features, including sulcal depth and curvature-based measures. Wright et al. (2014) utilized a non-linear model to predict fetal brain age based on curvature-based cortical folding measures and Namburete et al. (2015) developed a regression forest predictor. The abovementioned methods directly used pre-computed fetal cortical folding features for associating with the GA, whereas the ordinary deep CNN-based models have considered raw input images to “learn” parameterized feature extractors to minimize a task-specific objective function (e.g., mean square error function in brain age prediction). However, despite the superior performance of deep CNN-based brain age predictors based on their ability to build an expressive non-linear function without prior knowledge, the actual reasoning process for the prediction is hidden in the black-box nature of deep learning-based models. The interpretation of which features are extracted from the input MRI and how they affect the brain age prediction task remains an open problem. To the best of our knowledge, this is the first study to interpret deep-learning-based PAD using various global and regional cortical structural features.

Our results showed that the local surface area in all cerebral regions except for the paracentral and parahippocampal cortices and the whole cerebral volume was significantly increased in subjects with higher PAD. Significant correlations between these measures and the estimated PAD are consistent with a previous study (Hong et al., 2021) that showed a low predicted brain age for subjects with reduced brain size. Interestingly, our analysis of the cortical curvature and depth, which represent the shape of cortical folding, showed a positive correlation with the estimated PAD in specific regions, including some perisylvian regions and the insular cortex. Recent studies have shown that the perisylvian regions show pronounced age-related changes in fetal brains (Vasung et al., 2021)and that significant folding changes occur in the middle fetal stage at around 24–25 GW in the insular cortex (Ortinau et al., 2019). Thus, the estimated PAD might be affected by the complex patterning of cortical folding structures in specific regions during early fetal cortical development as well as global cerebral growth.

Sulcal folding patterns may be related to the patterning of cortical functional areas and are visible indicators of anatomical neuronal connections (Essen, 1997). Cortical areas do not develop independently but rather in relation to other functional areas with optimized white matter connections, and accordingly show the specific positions and sizes of these areas (O'leary et al., 2007). These aspects of early cortical arealization and organization may give rise to specific sulcal area patterns, which show the geometric and topological relationships of the sulcal folds. In humans, changes in complex sulcal patterns are difficult to detect by visual inspection. We found that the predicted brain age decreased in subjects with more atypical and disorganized area patterns of the sulcal folds. In particular, PAD was significantly associated with relative inter-sulcal areal relationships that characterize intrinsic sulcal patterns less affected by global factors such as overall brain size and shape. We suggest that the PAD estimated by our deep learning model can sensitively reflect variations in the interrelated arrangement and areal patterning of the sulcal folds.

Since cortical folds are not fully established in early and mid-gestation, it is challenging to define gyral parcel labels in unfolded cortical areas in early fetal brains. Thus, future studies are needed to improve the accuracy of cortical parcellation and regional analysis in early fetal stages. Furthermore, although the ventricular regions were highly predictive of brain age in previous studies (Shi et al., 2020; Hong et al., 2021), the features of the ventricle were not considered in this study. Future studies incorporating ventricle segmentation and volume quantification would be helpful for further understanding the brain structural features associated with brain age estimation.

In conclusion, we interpreted changes in PAD by identifying associations with fetal MRI-derived cortical structural features. Specifically, our statistical results show that the estimated PAD is correlated with not only size-related features (cortical volume and surface area) but also cortical folding measurements. Brain size, cortical folding shape (cortical curvature and sulcal depth) in specific regions, including the perisylvian areas, and sulcal area patterning affect the variance in brain age predicted by the deep learning-based model. These results allow us to better interpret the deep learning-based brain age prediction model by revealing what the model reflects when calculating brain age.

## Data availability statement

The data analyzed in this study is subject to the following licenses/restrictions: deidentified MRI data may be shared upon request after signing a data sharing agreement required by authors’ institution. Requests to access these datasets should be directed to KI, kiho.im@childrens.harvard.edu.

## Ethics statement

The studies involving humans were approved by Boston Children’s Hospital, Division of Newborn Medicine. The studies were conducted in accordance with the local legislation and institutional requirements. Written informed consent for participation in this study was provided by the participants’ legal guardians/next of kin.

## Author contributions

HK: Formal analysis, Methodology, Software, Visualization, Writing – original draft, Writing – review & editing. SY: Data curation, Methodology, Resources, Software, Writing – original draft. HY: Data curation, Methodology, Software, Validation, Writing – original draft. SJ: Data curation, Investigation, Resources, Writing – review & editing. AL: Resources, Software, Validation, Writing – review & editing. ML: Resources, Software, Validation, Writing – review & editing. PV: Resources, Software, Validation, Writing – review & editing. SD: Resources, Software, Validation, Writing – review & editing. PG: Writing – review & editing, Resources, Supervision. J-ML: Supervision, Validation, Writing – review & editing, Resources. KI: Formal analysis, Project administration, Supervision, Validation, Writing – original draft, Writing – review & editing, Funding acquisition, Resources.
