# Supplementary material for: The role of cortical structural variance in deep learning-based prediction of fetal brain age
Source: Front Neurosci. 2024 May 23;18:1411334. doi: 10.3389/fnins.2024.1411334 (PMC11153753; doi:10.3389/fnins.2024.1411334)
Supplement: Supplementary file 1 [file Table_1.DOCX]

Supplementary Material

# Supplementary Tables

**Table S1.** Statistical results for regression analysis of regional cortical measurements.

| **Index** | **Region** | **Sulcal area** | **Mean curvature** | **Sulcal depth** |
| --- | --- | --- | --- | --- |
| 1 | Middle frontal | 0.867 ± 0.293  (0.004*) | 0.104 ± 0.209  (0.619) | 0.038 ± 0.176  (0.828) |
| 2 | Cingulate cortex | 0.946 ± 0.303  (0.002*) | -0.008 ± 0.202  (0.967) | 0.018 ± 0.147  (0.905) |
| 3 | Cuneus | 0.726 ± 0.248  (0.004*) | 0.607 ± 0.235  (0.011*) | 0.229 ± 0.198  (0.249) |
| 4 | Precentral | 0.922 ± 0.287  (0.002*) | 1.022 ± 0.267  (0.0002*) | 0.388 ± 0.261  (0.140) |
| 5 | Fusiform | 0.605 ± 0.251  (0.018*) | 0.065 ± 0.239  (0.787) | 0.083 ± 0.180  (0.647) |
| 6 | Inferior parietal | 0.744 ± 0.226  (0.001*) | 0.102 ± 0.277  (0.714) | 0.008 ± 0.206  (0.968) |
| 7 | Inferior temporal | 0.551 ± 0.230  (0.018*) | 0.083 ± 0.196  (0.672) | 0.147 ± 0.164  (0.373) |
| 8 | Insula | 0.760 ± 0.301  (0.013*) | 0.646 ± 0.244  (0.009*) | 0.710 ± 0.259  (0.007 †) |
| 9 | Precuneus | 0.654 ± 0.267  (0.016*) | 0.509 ± 0.316  (0.110) | 0.461 ± 0.208  (0.029 †) |
| 10 | Lateral occipital | 0.694 ± 0.240  (0.005*) | 0.087 ± 0.211  (0.680) | 0.114 ± 0.161  (0.480) |
| 11 | Orbital frontal | 1.369 ± 0.282  (<0.0001*) | 0.117 ± 0.200  (0.562) | 0.198 ± 0.173  (0.255) |
| 12 | Lingual | 0.857 ± 0.242  (0.0006*) | 0.569 ± 0.297  (0.058) | 0.201 ± 0.175  (0.253) |
| 13 | Superior frontal | 0.807 ± 0.272  (0.004*) | 0.148 ± 0.277  (0.594) | -0.007 ± 0.174  (0.970) |
| 14 | Middle temporal | 0.785 ± 0.253  (0.003*) | 0.213 ± 0.283  (0.452) | 0.042 ± 0.186  (0.821) |
| 15 | Paracentral | 0.279 ± 0.235  (0.238) | 0.555 ± 0.234  (0.194) | 0.007 ± 0.177  (0.970) |
| 16 | Parahippocampal | 0.261 ± 0.177  (0.143) | 0.038 ± 0.194  (0.846) | 0.104 ± 0.143  (0.467) |
| 17 | Inferior frontal | 0.794 ± 0.238  (0.001*) | 0.742 ± 0.281  (0.009*) | 0.371 ± 0.241  (0.127) |
| 18 | Superior parietal | 0.573 ± 0.240  (0.019*) | 0.045 ± 0.265  (0.865) | -0.091 ± 0.196  (0.645) |
| 19 | Superior temporal | 0.632 ± 0.278  (0.025*) | 0.701 ± 0.279  (0.013*) | 0.768 ± 0.282  (0.008 †) |
| 20 | Supramarginal | 0.711 ± 0.281  (0.013*) | 1.195 ± 0.322  (0.0003*) | 0.605 ± 0.271  (0.028 †) |
| 21 | Postcentral | 0.624 ± 0.301  (0.041*) | 0.945 ± 0.234  (0.0001*) | 0.449 ± 0.276  (0.106) |

Data are represented as *β* ± *SE* (*P value*).

* FDR-corrected *P* < 0.05. †: Uncorrected *P* < 0.05.
